# Birth weight and risk of childhood solid tumors in Brazil: a record linkage between population-based data sets

**DOI:** 10.26633/RPSP.2017.14

**Published:** 2017-02-08

**Authors:** Neimar de Paula Silva, Rejane de Souza Reis, Rafael Garcia Cunha, Julio Fernando Oliveira, Cristina Fernanda da Silva de Lima, Maria Socorro Pombo-de-Oliveira, Marceli Oliveira Santos, Beatriz de Camargo

**Affiliations:** 1 Centro de Pesquisa Instituto Nacional de Câncer Rio de Janeiro, RJ Brazil

**Keywords:** Child health, neoplasms, birth weight, fetal development, registries, Brazil, Salud del niño, neoplasias, peso al nacer, desarrollo fetal, sistema de registros, Brasil

## Abstract

**Objective.:**

To analyze the relationship between the development of childhood solid tumors and 1) birth weight and 2) fetal growth, using two Brazilian population-based data sets.

**Methods.:**

*A case–cohort study was performed using two population-based data sets, and linkage between the Live Birth Information System* (Sistema de Informação sobre Nascidos Vivos, SINASC) *and 14 population-based cancer registries (PBCRs) was established. Four controls per case were chosen randomly from the SINASC data set. Tumors were classified as central nervous system (CNS), non-CNS embryonal, and other tumors (“miscellaneous”). Adjustments were made for potential confounders (maternal age, mode of delivery, maternal education, birth order, gestational age, sex, and geographic region). Odds ratios (ORs) with 95% confidence intervals (CIs) were computed using unconditional logistic regression analysis.*

**Results.:**

In a trend analysis, for every 500 g of additional birth weight, the crude OR was 1.12 (CI: 1.00–1.24) and the adjusted OR was 1.02 (CI: 0.90–1.16) for all tumors. For every 1 000 g of additional birth weight, the crude OR was 1.25 (CI: 1.00–1.55) and the adjusted OR was 1.04 (CI: 0.82–1.34) for all tumors. Among children diagnosed after reaching the age of 3 years, in the miscellaneous tumor category, the OR was significantly increased for every additional 500 g and 1 000 g of birth weight.

**Conclusions.:**

The study data suggested that increased birth weight was associated with childhood solid tumor development, especially among children more than 3 years old with “miscellaneous” tumors.

## RESUMEN

**Objetivo.:**

Analizar la relación entre la aparición de tumores sólidos en la niñez y 1) el peso al nacer y 2) el crecimiento fetal, a partir de dos conjuntos de datos poblacionales del Brasil.

**Métodos.:**

Se efectuó un estudio de casos en una cohorte a partir de dos conjuntos de datos poblacionales y se vinculó el sistema de información de nacidos vivos (Sistema de Informação sobre Nascidos Vivos, SINASC) con 14 registros oncológicos poblacionales. Se eligieron al azar cuatro controles por caso del conjunto de datos del SINASC. Los tumores se clasificaron en tres tipos: del sistema nervioso central (SNC), embrionarios ajenos al SNC y otros (“misceláneos”). Se hicieron ajustes en función de los posibles factores de confusión (edad materna, modalidad de parto, educación materna, orden de nacimiento, edad gestacional, sexo y región geográfica) y se calcularon las razones de posibilidad (OR) con un intervalo de confianza (IC) del 95 % mediante análisis de la regresión logística incondicional.

**Resultados.:**

En el análisis de las tendencias, se observó que, en todos los tumores, cada 500 g adicionales de peso al nacer la OR bruta fue de 1,12 (IC: 1,00-1,24) y la OR ajustada, de 1,02 (IC: 0,90-1,16), mientras que, cada 1 000 g adicionales, la OR bruta fue de 1,25 (IC: 1,00-1,55) y la OR ajustada, de 1,04 (IC: 0,82-1,34). En cuanto a los niños diagnosticados después de los 3 años de edad, en la categoría de tumores misceláneos, la OR fue significativamente más alta con cada 500 g y 1 000 g adicionales de peso al nacer.

**Conclusiones.:**

Los datos del estudio indican que el peso alto al nacer está asociado a la aparición de tumores sólidos en la niñez, especialmente de la categoría “misceláneos” y en los niños mayores de 3 años de edad.

Causes of childhood cancer are poorly understood. Research showing transplacental exposure to carcinogenic agents as a risk factor for cancer has led to the notion that important events associated with the development of embryonic tumors can occur in utero (1). Birth characteristics have also been reported as risk factors, based on underpowered studies. However, high birth weight and/or accelerated fetal growth have been associated with an increased risk of childhood cancer (2, 3). In one study, high birth weight was associated with childhood acute leukemia risk (4). Results for research on the association of birth characteristics with other types of tumors have been more difficult to analyze because of the low relative frequency of the different tumor types. Results have been inconclusive for central nervous system (CNS) cancers, lymphomas, and neuroblastoma (3, 5, 6). In a systematic review, high birth weight was associated with a significantly increased risk of Wilms tumor (7).

Fetal growth can be influenced by environmental and genetic factors (8). Circulating insulin-like growth factors (IGFs) are highly correlated with fetal growth and are suggested to play an important role in carcinogenesis (9). Accelerated growth associated with growth factors in normal as well as cancer cells needs to be explored.

Socioeconomic factors have been related to birth weight. Establishing the cutoff for high and low birth weight in different societies can be a challenge. Population-based studies are essential to describe epidemiological patterns that can provide clues for future studies.

The aim of this study was to analyze the relationship between the development of childhood solid tumors and 1) birth weight and 2) fetal growth, using two Brazilian population-based data sets.

## MATERIALS AND METHODS

### Data sources

Data were obtained from 14 population-based cancer registries (PBCRs) in Brazil. Cases among children with solid tumors born after 1999 and diagnosed between 2000 and 2010 were included (*n* = 566). The National Cancer Institute (*Instituto Nacional de Câncer*, INCA) (Rio de Janeiro) has promoted the establishment of cancer registries in various Brazilian cities, and there are now 25 PBCRs distributed across major cities in all regions of Brazil. A total of 60% met International Agency for Research on Cancer (IARC) (Lyon) standard criteria for data quality parameters, such as percentage of cases 1) confirmed microscopically (> 75%) and 2) collected only through death certificates (< 20%) (10).

Data for 2000 to 2010 from Brazil’s Live Birth Information System (*Sistema de Informação sobre Nascidos Vivos*, SINASC) corresponding to cases obtained from PBCRs for 14 cities in Brazil’s five regions (*n* = 5 824 824) were obtained. Multiple pregnancies (*n* = 112 739; 1.9%) were excluded. Although SINASC was implemented in 1990, the system went through a maturation process, becoming more reliable since 2000, and the coverage of procedures has gradually improved. The information has been extensively used to obtain health indicators, and to conduct epidemiological studies and health surveillance activities. Data for variables such as birth weight, newborn sex, hospital of birth, type of delivery, maternal age, and maternal education level are considered to have good completeness and reliability (11, 12). The PBCR cases were classified as CNS tumors (*n* = 162), neuroblastoma (*n* = 82), retinoblastoma (*n* = 37), renal tumors (*n* = 84), liver tumors (*n* = 13), soft tissue sarcomas (*n* = 46), germ cell tumors (*n* = 40), bone tumors (*n* = 11), carcinomas (*n* = 31), and non-specified tumors (*n* = 60), based on the criteria of the International Classification of Childhood Cancer, Third Edition (ICCC-3) (13).

### Data linkage

The PBCRs and SINASC do not have a unique identifier. Therefore, determining characteristics of cases at birth required probabilistic data linkage through variables present in both databases, used in different combinations for comparison and blocking (mother’s name, child’s sex, birth date, address, and child’s race), which was done using R software (RecordLinkage Package) (14). The data sources were thus combined using probabilistic algorithms to identify records related to unique individuals. The Record-Linkage Package SoundexBR algorithm was used for phonetic comparisons (15) and the levenshteinSim algorithm (14) was used to compare strings.

### Study design

A case–cohort study was performed to select cases and controls within the same total population at baseline. Four controls per case were chosen through systematic random sampling (*n* = 1 580) from the SINASC data source, ordered by birth year and sex. Increasing the ratio of controls to cases beyond four is considered unnecessary except when the effect of exposure is large (16). The information on birth weight and gestational age was obtained from SINASC. The variables evaluated were sex, race, birth weight, gestational age, mode of delivery, birth order, maternal age at child’s birth, maternal education, birth anomalies, prenatal visits, and geographic region.

Finally, the data were grouped by geographic region (North, Northeast, Middle West, Southeast, and South). These data, along with the Human Development Index (HDI) score, childhood cancer incidence and mortality rates, and birth weight categories for Brazil’s five different regions, are shown in Table 1.

### Statistical analysis

Unconditional logistic regression analysis was carried out using IBM SPSS Statistics for Windows version 21.0 (IBM Corp., Armonk, New York, United States) to evaluate the association between birth weight, fetal growth, and childhood solid tumors. Odds ratios (ORs) with 95% confidence intervals (CIs) for birth weight and fetal growth were estimated in a multivariate model. Adjustments were made using variables that were potential risk factors for childhood cancer, such as maternal age at delivery, mode of delivery, maternal education level, birth order, gestational age, sex, and geographic region, and associated with birth weight. The analysis of all tumors and for each specific group always included the total control group for comparison. Cases with birth weight for gestational age (BWGA) that were missing data (18 that were missing gestational age and one that was missing sex) were excluded. Birth weight was 1) evaluated as a continuous variable, with units of 500 g and 1 000 g; 2) evaluated as a categorical variable, with three levels, including the reference group with normal birth weight (2 500– 4 000 g); and 3) divided into five categories, with the reference group as normal birth weight (3 000–3 499 g). Sex, birth weight, and gestational age were used to classify infants by weight into the following gestational age categories: large for gestational age (LGA), defined as a birth weight above the sex- and gestational age–specific 90th percentile; small for gestational age (SGA), defined as a birth weight below the 10th percentile; and appropriate for gestational age (AGA), defined as a birth weight between the 10th and 90th percentiles. A large, published birth cohort of Brazilian population birth weight centiles was used to define these categories (17). Separate analyses were carried out for CNS tumors; non-CNS embryonal tumors (neuroblastoma, retinoblastoma, renal tumors, soft tissue sarcomas, hepatoblastoma, and germ cell tumors); and the miscellaneous group (carcinomas, bone tumors, and non-specified tumors). A weighted kappa statistic was used to test the agreement between birth weight per se and BWGA. The weighted kappa quantified the dimension of agreement beyond the expected level of agreement from chance alone. The values of this test ranged from 1 (perfect agreement), to 0 (no agreement), to −1 (perfect disagreement); values around 0.5 represent fair agreement (18).

**TABLE 1.** Childhood cancer incidence and mortality rates, birth weight categories, and Human Development Index (HDI) score, by geographic region, Brazil, 2016

					Birth weight (%)	
Geographic region	Incidence (AAIR^a^)	Mortality (AAMR^b^)	HDI score	< 2 500 g	2 500–4 000 g	> 4 000 g
North	105.95	45.57	0.690	7.6	86.4	5.7
Northeast	123.70	39.43	0.660	7.9	86.0	6.0
Middle West	157.93	43.31	0.732	8.3	87.1	4.5
Southeast	132.01	40.84	0.751	9.2	86.7	4.1
South	166.24	46.47	0.749	8.6	86.6	4.8

Sources: Brazilian Ministry of Health population-based cancer registries (PBCRs) (http://www2.inca.gov.br/wps/wcn/connect/estatisticas/site/home/rcbp/); Institute of Geography and Statistics (IBGE) (www.ibge.gov.br/); Live Birth Information System (SINASC) (http://datasus.saude.gov.br/sistemas-e-aplicativos/eventos-v/sinasc-sistema-de-informacoes-de-nascidos-vivos); and Mortality Information System (SIM) (http://www.datasus.gov.br/catalogo/sim.htm).

a Age-adjusted incidence rate (median) of neoplasms per million children (0–14 years old).

b Age-adjusted mortality rate of neoplasms per million children (0–14 years old).

### Ethical considerations

All data were kept strictly confidential to ensure anonymity. The study was approved by INCA’s Research Ethics Committee (ref# 13596513.7.0000.5274).

## RESULTS

Linkage between the two sources identified 395 cases (69.8% of all PBCR cases). After excluding three cases that were missing BWGA, the cases were classified into CNS tumors (*n* = 127), neuroblastoma (*n* = 65), retinoblastoma (*n* = 28), renal tumors (*n* = 61), liver tumors (*n* = 6), soft tissue sarcomas (*n* = 29), germ cell tumors (*n* = 32), bone tumors (*n* = 5), carcinomas (*n* = 8), and non-specified tumors (*n* = 31). Linkage identified 74%–80% of CNS tumors, neuroblastoma, retinoblastoma, renal tumors, soft tissue sarcomas, and germ cell tumors; 45% of hepatoblastomas, bone tumors, and non-specified tumors; and only 26% of carcinomas.

The distribution patterns of the children included in the study sample are shown in Table 2. Birth anomalies were described as follows, based on the criteria of the 10th revision of the International Statistical Classification of Diseases and Related Health Problems (ICD-10): talipes equinovarus (*n* = 1), polydactyly (*n* = 1), syndactyly (*n* = 1), other congenital anomaly of the foot (*n* = 2), Down syndrome (*n* = 3), macrocephaly (*n* = 1), unspecified syndrome (*n* = 1), spina bifida (*n* = 1), unspecified brain abnormality (*n* = 1), congenital anomaly of male genital tract (*n* = 1), unspecified head and neck abnormality (*n* = 2), and multiple malformations (*n* = 1). There were minor differences among the mean birth weights for each tumor type and controls (*P* = 0.649). The mean birth weight calculated was 3 261 g (CI: 3 176–3 346 g) for CNS tumors; 3 260 g (CI: 3 154–3 366 g) for neuroblastomas; 3 287 g (CI: 3 101– 3 472 g) for retinoblastomas; 3 211 g (CI: 3089–3 334 g) for renal tumors; 3 131 g (CI: 2 537–3 724 g) for liver tumors; 3 618 g (CI: 3 085–4 151 g) for bone tumors; 3 307 g (CI: 3 111–3 504 g) for soft tissue sarcoma; 3 275 g (CI: 3 130–3 420 g) for germ cell tumors; 3 311 g (CI: 2 987–3 634 g) for carcinomas; 3 203 g (CI:3 009–3 396 g) for non-specified tumors; and 3 204 g (CI: 3 178–3 229 g) for the control group. Mean birth weight calculated by geographic region was 3 220 g (CI: 3 158–3 282 g) for the North region; 3 252 g (CI: 3 211–3 293 g) for the North-east; 3 163 g (CI: 3 117–3 208 g) for the Southeast; 3 212 g (CI: 3 166–3 257 g) for the South; and 3 253 g (CI: 3 172–3 333 g) for the Middle West. Among the case group, 77.4% of infants with SGA had an adequate birth weight (2 500–4 000 g). Among the control group, 63.7% of infants with SGA had an adequate birth weight (2 500–4 000 g). Overall, 66.3% of infants with SGA had an adequate birth weight (2 500–4 000 g). Tests were then run to determine if birth weight per se and BWGA were related. Overall, agreement between the two measures was moderate, as shown by a weighted kappa of 0.56 (CI: 0.53–0.59). There was no association between perinatal and demographic variables, despite a slight difference in both the control and case groups for number of prenatal visits and in the control group for maternal age. In the control group, males were heavier than females (*P* < 0.001) (Table 3). Crude and adjusted ORs for birth weight and fetal growth are shown in Table 4. In trend analysis, for every 500 g of additional birth weight, the crude OR was 1.12 (CI: 1.00–1.24) and the adjusted OR was 1.02 (CI: 0.90– 1.16). For every 1 000 g of additional birth weight, the crude OR was 1.25 (CI: 1.00–1.55) and the adjusted OR was 1.04 (CI: 0.82–1.34). For both the CNS and non-CNS embryonal tumor groups, similar results were seen. For the miscellaneous group, a risk of 1.17 (CI: 0.84–1.63) and 1.37 (CI: 0.71–2.66) was observed for each increase of 500 g and 1 000 g in weight at birth respectively.

Tests were also run to determine if the association with birth weight was significantly different when the diagnosis was made in children before or after they had reached 3 years of age. In the miscellaneous group, there was a significant (twofold) increase in risk for every 500 g of additional birth weight (adjusted OR = 1.78; CI: 1.02–3.14) in children who were diagnosed after reaching age 3.

The risk association between pediatric tumors, fetal growth, and birth weight, by age of diagnosis, is shown in Table 5. A threefold increase in risk was observed for every 1 000 g increase in weight (adjusted OR = 3.20; CI: 1.03–9.87).

## DISCUSSION

Birth weight has been associated with childhood cancer risk in the literature. High birth weight has also been implicated as a risk factor for adult cancers (breast, lung, colorectal, and prostate) (19). Several reports show a relationship between high birth weight and risk of leukemia (4). Several factors are related to birth weight, including maternal diet, gestational diabetes, maternal age, air pollution, and cigarette smoking. An increase in mean birth weight related to changes in socio-demographic factors has occurred in several developed countries (20). Lower socioeconomic strata have been associated with lower birth weight more often than higher social classes, and lower birth weight has been considered a marker of lower social development. Therefore, the higher rates of low birth weight in the more developed regions in Brazil with higher HDI scores (South and Southeast), as seen in Table 1, may be more associated with the availability of perinatal care services than with social conditions (21). Older maternal age has also been observed in more developed areas, associated with low birth weight (22). In the current study, mean birth weight was lower in the South and Southeast regions of Brazil. The authors suggest that this outcome may be related to older maternal age. Race can also be a factor, and Brazil has one of the most racially admixed populations worldwide. In one study in Brazil, preterm births and low birth weight were significantly higher among infants of African ancestry than among those of solely European ancestry (23). In the SINASC data set, race was considered an adverse variable, with inconsistent and missing information (12). However, in the current study, race was not related to birth weight.

**TABLE 2.** Socio-demographic characteristics of childhood cancer cases and controls, by type of tumor, Brazil, 2000–2010

							Type of tumor		
Characteristic	Controls	Cases	P	CNS^a^	P	Non-CNS embryonal^b^	P	Miscellaneous^c^	P
	No. (%)	No. (%)		No. (%)		No. (%)		No. (%)	
Gender									
Male	798 (51.0)	230 (58.7)		80 (63.0)		126 (57.0)		24 (54.5)	
Female	766 (49.0)	162 (41.3)	0.007	47 (37.0)	0.012	95 (43.0)	0.097	20 (45.5)	0.645
Race									
White	734 (46.9)	173 (44.1)		56 (44.1)		103 (46.6)		14 (31.8)	
Non-white	729 (46.6)	201 (51.3)		67 (52.7)		108 (48.9)		26 (59.1)	
Missing data	101 (6.5)	18 (4.6)	0.153	4 (3.1)	0.179	10 (4.5)	0.486	4 (9.1)	0.137
Geographic region									
North	204 (13.0)	49 (12.5)		16 (12.6)		25 (11.3)		8 (18.2)
Northeast	506 (32.4)	127 (32.4)		37 (29.1)		77 (34.8)		13 (29.5)
South	348 (22.3)	87 (22.2)		30 (23.6)		47 (21.3)		10 (22.7)	
Southeast	427 (27.3)	109 (27.8)		37 (29.1)		63 (28.5)		9 (20.5)	
Midwest	79 (5.1)	20 (5.1)	0.999	7 (5.5)	0.955	9 (4.1)	0.862	4 (9.1)	0.593
Birth order									
First	540 (34.5)	148 (37.7)		44 (34.6)		85 (38.5)		19 (43.2)	
Second or higher	899 (57.5)	216 (55.1)		72 (56.7)		123 (55.7)		21 (47.7)	
Missing data	125 (8.0)	28 (7.1)	0.272	11(8.7)	0.971	13 (5.9)	0.331	4 (9.1)	0.433
Maternal age (years)									
< 25	821 (52.5)	199 (50.8)		67 (52.8)		105 (47.5)		27 (61.4)	
25–35	635 (40.6)	166 (42.3)		50 (39.4)		103 (46.6)		13 (29.5)	
> 35	104 (6.7)	27 (6.9)		10 (7.9)		13 (5.9)		4 (9.1)	
Missing data	4 (0.3)	–	0.804	–	0.817	–	0.274	–	0.458
Maternal education (years)									
< 3	238 (15.2)	41 (10.5)		10 (7.9)		23 (10.4)		8 (18.2)	
4–11	1 065 (68.1)	261 (66.6)		82 (64.6)		151 (68.3)		28 (63.6)	
≥ 12	220 (14.1)	79 (20.1)		31 (24.4)		42 (19.0)		6 (13.6)	
Missing data	41 (2.6)	11 (2.8)	0.002	4 (3.2)	0.002	5 (2.3)	0.129	2 (4.5)	0.836
Gestational age (weeks)									
< 37	91 (5.8)	16 (4.1)		7 (5.5)		5 (2.3)		4 (9.1)	
37–41	1 453 (92.9)	374 (95.4)		119 (93.7)		215 (97.3)		40 (90.9)	
> 41	20 (1.3)	2 (0.5)	0.133	1 (0.8)	0.141	1 (0.5)	0.001	–	0.407

***Source:*** Prepared by the authors based on the study results.

a Central nervous system.

b Neuroblastoma, retinoblastoma, renal tumors, soft tissue sarcomas, hepatoblastoma, and germ cell tumors.

c Carcinomas, bone tumors, and non-specified tumors.

While errors in birth weight have occurred in the SINASC neonatal registry, the SINASC data have an intraclass correlation coefficient of 0.57, suggesting that socioeconomic status can be used to evaluate risk (24). Previous data for the birth weight variable countrywide, except in the northern state of Acre, were considered excellent (12). Mean birth weights at most gestational ages in this research were similar to other findings recorded in the literature. However, a higher frequency of SGA was seen among both the case and control groups in this study (15.8% and 17.5% respectively) compared with the range reported in the literature (8%–11%) (25–28).

Several authors have suggested that BWGA is an indicator of fetal growth and a better predictor than birth weight per se for risk of several tumor types (4, 5). Birth weight per se and BWGA may share environmental as well as genetic determinants. In turn, levels of hormones and other growth factors may play a role in the development of leukemia and CNS tumors (29). Wilms tumor is strongly correlated with overgrowth syndromes such as Perlman, Simpson–Golabi–Behmel, and Beckwith–Wiedemann syndromes, and also has been associated with high birth weight independent of coexisting congenital abnormalities (7). However, in the 61 cancer patients studied in this research, there were no documented cases of overgrowth syndromes and the risk could not be related to birth weight.

**TABLE 3:** Perinatal and demographic variables by birth weight of childhood cancer cases and controls, Brazil, 2000–2010

		Controls				Cases		
		Birth weight (g)				Birth weight (g)		
Variable^a^	< 2 500	2 500–4 000	> 4 000	P	< 2 500	2 500–4 000	> 4 000	P
	No. (%)	No. (%)	No. (%)		No. (%)	No. (%)	No. (%)	
Gender								
Female	57 (49.1)	694 (50.4)	15 (21.4)		7 (43.8)	150 (42.4)	5 (22.7)	
Male	59 (50.9)	684 (49.6)	55 (78.6)	< 0.0001	9 (56.3)	204 (57.6)	17 (77.3)	0.189
Race								
White	50 (46.3)	646 (50.2)	38 (55.9)		10 (71.4)	156 (45.9)	7 (35.0)	
Non-white	58 (53.7)	641 (49.8)	30 (44.1)	0.464	4 (28.6)	184 (54.1)	13 (65.0)	0.101
Geographic region								
North	16 (13.8)	175 (12.7)	13 (18.6)		1 (6.3)	43 (12.1)	5 (22.7)	
Northeast	33 (28.4)	447 (32.4)	26 (37.1)		7 (43.8)	113 (31.9)	7 (31.8)	
Southeast	36 (31.0)	376 (27.3)	15 (21.4)		4 (25.0)	101 (28.5)	4 (18.2)	
South	27 (23.0)	307 (22.3)	14 (20.0)		4 (25.0)	77 (21.8)	6 (27.3)	
Middle West	4 (3.4)	73 (5.3)	2 (2.9)	0.65	–	20 (5.6)	–	0.426
Birth order								
First	39 (36.1)	485 (38.3)	16 (24.6)		6 (37.5)	136 (41.3)	6 (31.6)	
Second or higher	69 (63.9)	781 (61.7)	49 (75.4)	0.08	10 (62.5)	193 (58.7)	13 (68.4)	0.678
Maternal age (years)								
< 25	58 (50.0)	734 (53.4)	29 (41.4)		8 (50.0)	179 (50.6)	12 (54.5)	
25–35	48 (41.4)	547 (39.8)	40 (57.1)		5 (31.3)	153 (43.2)	8 (36.4)	
> 35	10 (8.6)	93 (6.8)	1 (1.4)	0.033	3 (18.8)	22 (6.2)	2 (9.1)	0.489
Maternal education (years)								
≤ 3	19 (17.0)	205 (15.3)	14 (20.6)		3 (18.8)	36 (10.4)	2 (10.5)	
4–11	72 (64.3)	946 (70.4)	47 (69.1)		12 (75.0)	237 (68.5)	12 (63.2)	
≥ 12	21 (18.8)	192 (14.3)	7 (10.3)	0.386	1 (6.3)	73 (21.1)	5 (26.3)	0.475
Mode of delivery								
Vaginal	67 (58.3)	828 (60.2)	26 (37.1)		10 (62.5)	172 (48.6)	8 (36.4)	
Cesarean	48 (41.7)	548 (39.8)	44 (62.9)	0.001	6 (37.5)	182 (51.4)	14 (63.6)	0.279
Birth anomalies								
No	111 (100)	1 311 (99.5)	69 (100)		15 (100)	336 (97.4)	20 (95.2)	
Yes	–	6 (0.5)	–	0.663	–	9 (2.6)	1 (4.8)	0.576
Prenatal visits								
≤ 3	24 (21.4)	153 (11.2)	9 (13.0)		4 (26.7)	34 (9.8)	1 (4.8)	
4–6	35 (31.3)	471 (34.6)	22 (31.9)		8 (53.3)	106 (30.5)	4 (19.0)	
≥ 7	53 (47.3)	737 (54.2)	38 (55.1)	0.035	3 (20.0)	208 (59.8)	16 (76.2)	0.012

***Source:*** Prepared by the authors based on the study results.

a Missing data: race (*n* = 119); birth order (*n* = 153); maternal age (*n* = 4); maternal education (*n* = 52); mode of delivery (*n* = 3); birth anomalies (*n* = 78); perinatal care (*n* = 30).

High birth weight has been reported as a risk factor for bone tumors (30). Among the five cases of bone cancer included in this study, three had birth weights of more than 3 500 g. Carcinoma was not associated with high birth weight (31). There were 31 cases of non-cancers, and the inclusion criteria for that group included all possible diagnoses. Among the cases included in the PBCRs, a high incidence of non-specified tumors (a median of seven cases per million) has previously been reported (32). Non-specified tumors are probably misclassified as uncertain diagnoses and may correspond to several histological subtypes. Unfortunately, the finding in this study indicating an increasing risk of cancer with higher birth weight only suggests an increased risk of solid tumors (i.e., no association was determined for any specific type).

Overall, this study found a 4% increase in childhood solid tumors for every 1000 g of additional birth weight. No differences were seen for either CNS or non-CNS embryonal tumors. Nevertheless, because it is well known that hepatoblastoma, which was included in the non-CNS embryonal tumor group, is associated with low birth weight, the six cases of hepatoblastoma were excluded from the study. For the miscellaneous group, a risk of 1.37 (CI: 0.71–2.66) was observed for each additional 1 000 g of birth weight. When data were stratified based on age at diagnosis, birth weight was associated with the miscellaneous category, with a risk of 1.27 (CI: 0.82–1.99) for each increase of 1 000 g, for children 3 or more years old. Children less than 3 years old at diagnosis who were small for their gestational age had a 45% higher risk of CNS tumors, although the difference was not significant (OR = 1.45; CI: 0.81–2.61). In a recent pooled analysis, higher birth weight was associated with non-leukemia cancer diagnosed at or after the age of 3 years (8). In another report, when disease was diagnosed before the age of 2 years, there was an increased risk for all tumors with higher birth weight (33).

**TABLE 4:** Risk association of pediatric tumors, fetal growth, and birth weight, Brazil, 2000–2010

Type of tumor	Crude OR^a^(CI^b^)	P	Adjusted OR (CI)	P
All (*n* = 392)				
Birth weight^c^				
Per 500 g increase	1.12 (1.00–1.24)		1.02 (0.90–1.16)	
Per 1 000 g increase	1.25 (1.00–1.55)	0.048	1.04 (0.82–1.34)	0.098
< 2 500 g	0.53 (0.31–0.91)	0.021	0.58 (0.31–1.07)	0.089
2 500–4 000 g	1.00		1.00	
> 4 000 g	1.22 (0.74–2.00)	0.857	0.93 (0.52–1.66)	0.539
Fetal growth^d^				
SGA^e^	0.89 (0.65–1.20)	0.457	0.99 (0.72–1.36)	0.467
AGA^f^	1.00		1.00	
LGA^g^	1.05 (0.64–1.73)	0.828	0.93 (0.53–1.63)	0.765
CNS^h^(*n* = 127)				
Birth weight^c^				
Per 500 g increase	1.12 (0.94–1.34)		1.00 (0.82–1.22)	
Per 1 000 g increase	1.25 (0.88–1.79)	0.210	1.01 (0.68–1.50)	0.350
< 2 500 g	0.63 (0.27–1.46)	0.273	0.56 (0.21–1.48)	0.263
2 500–4 000 g	1.00		1.00	
> 4 000 g	1.39 (0.65–2.96)	0.501	0.90 (0.34–2.34)	0.456
Fetal growth^d^				
SGA	0.85 (0.51–1.41)	0.539	1.05 (0.62–1.77)	0.683
AGA	1.00		1.00	
LGA	1.41 (0.68–2.90)	0.346	1.15 (0.48–2.76)	0.403
Non-CNS embryonal^i^ (*n* = 221)				
Birth weight^c^				
Per 500 g increase	1.11 (0.96–1.27)		1.00 (0.85–1.18)	
Per 1 000 g increase	1.22 (0.93–1.62)	0.090	1.01 (0.73–1.39)	0.345
< 2 500 g	0.47 (0.22–0.97)	0.038	0.69 (0.30–1.53)	0.159
2 500–4 000 g	1.00		1.00	
> 4 000 g	1.07 (0.55–2.05)	0.659	0.89 (0.43–1.86)	0.486
Fetal growth^d^				
SGA	0.91 (0.62–1.33)	0.645	0.97 (0.65–1.45)	0.735
AGA	1.00		1.00	
LGA	0.88 (0.45–1.74)	0.733	0.76 (0.35–1.62)	0.765
Miscellaneous^j^ (*n* = 44)				
Birth weight^c^				
Per 500 g increase	1.14 (0.85–1.53)		1.17 (0.84–1.63)	
Per 1 000 g increase	1.30 (0.72–2.34)	0.193	1.37 (0.71–2.66)	0.153
< 2 500 g	0.60 (0.14–2.55)	0.489	0.36 (0.07–1.91)	0.439
2 500–4 000 g	1.00		1.00	
> 4 000 g	1.51 (0.45–5.02)	0.731	1.32 (0.30–5.84)	0.806
Fetal growth^d^				
SGA	0.88 (0.39–2.02)	0.778	0.90 (0.36–2.22)	0.789
AGA	1.00		1.00	
LGA	0.88 (0.20–3.76)	0.883	1.21 (0.27–5.25)	0.867

***Source:*** Prepared by the authors based on the study results.

a Odds ratio.

b 95% confidence interval.

c Adjusted by maternal age at birth, mode of delivery, maternal education, birth order, geographic region, gestational age, and sex.

d Adjusted by maternal age at birth, mode of delivery, maternal education, birth order, and geographic region.

e Small for gestational age.

f Appropriate for gestational age.

g Large for gestational age.

h Central nervous system.

i Neuroblastoma, retinoblastoma, renal tumors, soft tissue sarcomas, hepatoblastoma, and germ cell tumors.

j Carcinomas, bone tumors, and non-specified tumors.

In a nested case–control study from Brazilian hospital-based data, mean birth weight for cases was 3 320 g, slightly higher than the mean birth weight of the cases in this study (3 259 g).

## Strengths and limitations

The main strength of this study was the use of population-based data rather than self-reported data (i.e., data reported by patients after the determination of their disease status), which prevented recall bias issues. In addition, controls and cases were chosen from the same birth certificate database, so selection and information bias are also unlikely. Finally, the study’s main data source (the SINASC database) is recognized as being of good quality, with complete and consistent information (12). Unfortunately, the study only assessed regional SINASC data that included the cities with the 14 PBCRs that supplied the other data set, which eliminated about one-third of cases, and emigration data were not available. The content of the PBCRs is also considered to be of good quality. PBCRs have significantly improved the quality of their data in the past decade in middle-income countries (34). The Brazilian Health Ministry has launched several initiatives to improve the quality of information in Brazil’s PBCRs. Evidence of improvement can be seen in an assessment by the IARC (35).

This study also had several limitations. First, the SINASC data on birth characteristics have only been available since 2000. Therefore, selected cases were obtained from the cohort born after 1999 and diagnosed from 2000–2010, limiting the analysis to data from children 0 to 10 years old. Second, the small size of the data set generated a low number of different tumor types, making it impossible to analyze by tumor type. Third, information on other potential confounders, such as infectious diseases and environmental exposures (e.g., maternal smoking), was not available. Finally, maternal education was used as proxy for socioeconomic status, which is not optimum, but no other adequate variable was available.

**TABLE 5.** Risk association of pediatric tumors, fetal growth, and birth weight, by age of diagnosis, Brazil, 2000–2010

		Diagnosed at < 3 years old				Diagnosed at ≥ 3 years old		
Type of tumor	Crude OR^a^(CI^b^)	P	Adjusted OR (CI)	P	Crude OR (CI)	P	Adjusted OR (CI)	P
All (*n* = 392)								
Birth weight^c^								
Per 500 g increase	1.09 (0.96–1.24)		1.01 (0.88–1.17)		1.17 (0.97–1.41)		1.12 (0.90–1.41)	
Per 1 000 g increase	1.20 (0.93–1.53)	0.102	1.03 (0.77–1.36)	0.365	1.37 (0.93–2.00)	0.100	1.27 (0.82–1.99)	0.099
< 2 500 g	0.71 (0.40–1.24)	0.305	0.71 (0.37–1.37)	0.309	0.11 (0.01–0.82)	0.035	0.17 (0.02–1.32)	0.091
2 500–4 000 g	1.00		1.00		1.00		1.00	
> 4 000 g	1.18 (0.66–2.09)	0.215	0.85 (0.43–1.66)	0.338	1.32 (0.59–2.95)	0.298	1.16 (0.44–3.02)	0.729
Fetal growth^d^								
SGA^e^	1.00 (0.71–1.4)	0.398	1.14 (0.80–1.63)	0.419	0.62 (0.34–1.13)	0.098	0.67 (0.36–1.23)	0.309
AGA^f^	1.00		1.00		1.00		1.00
LGA^g^	1.00 (0.55–1.8)	0.432	0.91 (0.47–1.76)	0.737	1.18 (0.53–2.63)	0.165	0.95 (0.37–2.45)	0.905
CNS^h^ (*n* = 127)								
Birth weight^c^								
Per 500 g increase	1.11 (0.90–1.37)		1.00 (0.79–1.27)		1.13 (0.83–1.54)		1.00 (0.70–1.44)	
Per 1 000 g increase	1.24 (0.81–1.89)	0.101	1.01 (0.63–1.60)	0.379	1.28 (0.69–2.38)	0.126	1.01 (0.49–2.09)	0.652
< 2 500 g	0.95 (0.40–2.22)	0.862	0.83 (0.30–2.33)	0.728	–	–	–	–
2 500–4 000 g	1.00		1.00		1.00		1.00	
> 4 000 g	1.57 (0.66–3.74)	0.301	1.07 (0.36–3.11)	0.875	1.03 (0.24–4.38)	0.689	0.43 (0.13–1.42)	0.932
Fetal growth^d^								
SGA	1.11 (0.63–1.95)	0.295	1.45 (0.81–2.61)	0.242	0.38 (0.11–1.24)	0.097	0.48 (0.06–3.60)	0.194
AGA	1.00		1.00		1.00		1.00
LGA	1.70 (0.75–3.83)	0.297	1.61 (0.61–4.22)	0.350	0.88 (0.20–3.76)	0.297	0.56 (0.07–4.27)	0.467
Non-CNS embryonal^i^(*n* = 221)								
Birth weight^c^								
Per 500 g increase	1.12 (0.95–1.31)		1.02 (0.85–1.23)		1.07 (0.83–1.39)		1.09 (0.80–1.47)	
Per 1 000 g increase	1.24 (0.91–1.71)	0.189	1.05 (0.73–1.52)	0.354	1.16 (0.69–1.95)	0.155	1.18 (0.64–2.16)	0.161
< 2 500 g	0.90 (0.58–1.39)	0.456	0.73 (0.30–1.76)	0.479	0.23 (0.03–1.73)	0.155	0.47 (0.06–3.56)	0.468
2 500–4 000 g	1.00		1.00		1.00		1.00	
> 4 000 g	0.70 (0.30–1.64)	0.492	0.73 (0.30–1.74)	0.270	1.57 (0.55–4.48)	0.576	1.50 (0.43–5.22)	0.602
Fetal growth^d^								
SGA	0.54 (0.25–1.19)	0.315	0.97 (0.61–1.54)	0.856	0.95 (0.45–1.97)	0.864	0.95 (0.45–2.01)	0.880
AGA	1.00		1.00		1.00		1.00	
LGA	0.90 (0.40–2.00)	0.354	0.60 (0.23–1.54)	0.269	1.48 (0.51–4.23)	0.658	1.30 (0.38–4.37)	0.707
Miscellaneous^j^ (*n* = 44)								
Birth weight^c^								
Per 500 g increase	0.91 (0.64–1.30)		0.91 (0.61–1.35)		1.67 (1.03–2.69)		1.78 (1.02–3.14)	
Per 1 000 g increase	0.83 (0.41–1.69)	0.617	0.83 (0.38–1.83)	0.655	2.79 (1.07–7.29)	0.036	3.20 (1.03–9.87)	0.043
< 2 500 g	1.03 (0.24–4.43)	0.689	0.45 (0.07–2.69)	0.381	–		–	–
2 500–4 000 g	1.00		1.00		1.00		1.00	
> 4 000 g	1.71 (0.39–7.40)	0.650	0.95 (0.11–7.76)	0.864	1.23 (0.16–9.41)	0.891	1.71 (0.21–13.70)	0.748
Fetal growth^d^								
SGA	1.33 (0.53–3.35)	0.565	1.39 (0.49–3.91)	0.630	0.29 (0.03–2.25)	0.315	0.33 (0.04–2.56)	0.321
AGA	1.00		1.00		1.00		1.00	
LGA	0.77 (0.10–5.86)	0.698	1.06 (0.13–8.46)	0.931	1.03 (0.13–7.95)	0.929	1.21 (0.15–9.62)	0.890

***Source:*** Prepared by the authors based on the study results.

a Odds ratio.

b 95% confidence interval.

c Adjusted by maternal age at birth, mode of delivery, maternal education, birth order, geographic region, gestational age, and sex.

d Adjusted by maternal age at birth, mode of delivery, maternal education, birth order, and geographic region.

e Small for gestational age.

f Appropriate for gestational age.

g Large for gestational age.

h Central nervous system.

i Neuroblastoma, retinoblastoma, renal tumors, soft tissue sarcomas, hepatoblastoma, and germ cell tumors.

j Carcinomas, bone tumors, and non-specified tumors.

## Conclusions

The findings of this study suggest that increased birth weight is associated with the development of childhood solid tumors, especially among children more than 3 years old at diagnosis, with tumors classified as “miscellaneous.” Birth weight by gestational age was not associated with this outcome. In the study’s heterogeneous population, birth weight was a difficult variable to interpret. Birth weight according to ethnic, socioeconomic, and perinatal care differences is still challenging to analyze. Larger samples are necessary. However, the Brazilian Live Birth Information System (SINASC) cohort used in this study only dated back to 2000, and the country’s PBCRs only had updated information until 2010. In the near future, as the scope of both data sources grows, it will be possible to obtain larger samples.

## Acknowledgments.

The authors are grateful to all of the coordinators of the PBCRs and regional SINASCs in Brazil who contributed the data sets that made this work possible.

## Conflicts of interest

None.

## Funding.

NPS has a scholarship from CAPES/MS (Ministry of Education Coordination for the Improvement of Higher Education Personnel/Ministry of Health) (Brasília). BDC has a scholar grant from the National Council for Scientific and Technological Development (*Conselho Nacional de Desenvolvimento Científico e Tecnológico*, CNPq) (Brasília) (#306291/2014-2) and the Foundation for Support of Research in the State of Rio de Janeiro (*Fundação de Amparo à Pesquisa do Estado do Rio de Janeiro*, FAPERJ) (Rio de Janeiro) (#212989-2016).

## Disclaimer.

Authors hold sole responsibility for the views expressed in the manuscript, which may not necessarily reflect the opinion or policy of the *RPSP/PAJPH* or the Pan American Health Organization (PAHO).
